# Supplementary material for: Lifestyle and Progression to Type 2 Diabetes in a Cohort of Workers with Prediabetes
Source: Nutrients. 2020 May 25;12(5):1538. doi: 10.3390/nu12051538 (PMC7284825; doi:10.3390/nu12051538)
Supplement: Supplementary file 1 [file nutrients-12-01538-s001.pdf]

Supplementary Material

Table S1. Association between final glycaemic status after 5 years of follow-up and studied variables (n=23,293).

Variables	Crude Odds Ratios		Adjusted Model	
	**Normalized OR (CI95%)**	**Progressed OR (95%CI)**	**Normalized aOR (CI95%)**	**Progressed aOR (95%CI)**
**Age**	0.98 (0.97-0.98)***	1.05 (1.04-1.05)***	0.99 (0.99-0.99)***	1.11 (1.10-1.12)***
**Male**	0.80 (0.75-0.85)***	0.90 (0.83-0.97)**	0.95 (0.88-1.03)	1.16 (1.01-1.34)*
**Blue-collar**	0.98 (0.92-1.05)	1.07 (0.98-1.16)	1.02 (0.94-1.10)	0.74 (0.64-0.86)***
**BMI**	0.84 (0.83-0.85)***	1.55 (1.53-1.57)***	0.95 (0.94-0.96)***	1.76 (1.72-1.81)***
**SBP**	0.99 (0.98-0.99)***	1.02 (1.02-1.03)***	1.00 (1.00-1.00)	1.00 (1.00-1.00)
**TG**	0.99 (0.99-0.99)***	1.00 (1.00-1.00)***	1.00 (1.00-1.00)***	1.00 (1.00-1.00)
**HbA1c ≤ 6%**	1.17 (1.09-1.25)***	0.02 (0.02-0.03)***	1.05 (0.97-1.13)	0.02 (0.01-0.02)***
**Diet (daily fruits & vegetables)**	3.53 (3.32-3.76)***	0.18 (0.16-0.20)***	1.35 (1.24-1.47)***	1.15 (0.94-1.40)
**PA (≥150 min/week)**	4.89 (4.59-5.21)***	0.03 (0.02-0.03)***	2.96 (2.68-3.26)***	0.17 (0.12-0.25)***
**Smoking**				
**Former smoker**	0.59 (0.54-0.64)***	1.03 (0.94-1.13)	0.58 (0.53-0.64)***	1.92 (1.61-2.29)***
**Current smoker**	0.96 (0.90-1.02)	0.68 (0.63-0.73)***	0.89 (0.83-0.95)**	1.03 (0.90-1.18)

Multinomial logistic regression analysis with persistence in prediabetes as a reference group. OR: Odds Ratio; CI: Confidence Interval *p<0.05; **p<0.01; ***p<0.001
BMI, Body Mass Index; SBP, Systolic Blood Pressure; TG, Triglycerides; HbA1c, Glycated Hemoglobin; PA, Physical Activity.
